# PAEAN: Pain in Aging, Educational Assessment of Need - An Interprofessional Collaboration

**DOI:** 10.1093/geroni/igab046.3059

**Published:** 2021-12-17

**Authors:** Beth Hogans, Bernadette Siaton, Laura Frey-Law, Lana Brown, Chris Herndon, Luis Buenaver, Les Katzel, Patricia Thomas

**Affiliations:** 1 VAMHCS, Baltimore, Maryland, United States; 2 University of Maryland School of Medicine, Baltimore, Maryland, United States; 3 University of Iowa Health Care, Carver College of Medicine, Iowa City, Iowa, United States; 4 Central Arkansas Veterans Healthcare System, Little Rock, Arkansas, United States; 5 School of Pharmacy Southern Illinois University Edwardsville, Edwardsville, Illinois, United States; 6 Johns Hopkins University, Baltimore, Maryland, United States; 7 Case Western Reserve School of Medicine, Cleveland, Ohio, United States

## Abstract

Pain is prevalent in older adults limiting independence directly and through comorbidity-related effects on functional domains such as mobility, well-being, sleep, productivity, and oligo-pharmacy. Improved outcomes for older adults with pain depends on provider knowledge and competence; concomitantly, Veterans, women, and others at socioeconomic disadvantage may face increased pain, comorbidities, and complications of treatments. Previous guidance for educational programs, from pre-licensure to post-graduate training, in geriatrics and pain have focused on expert opinion, whereas an evidence-based approach is preferred. Our working group is conducting a structured needs assessment regarding comorbidities of common pain-associated conditions in older adults. Methods: To capture expertise in medicine, nursing, pharmacy, clinical psychology, and physical therapy, we extended an open invitation to members of the VA Geriatric Research, Education, Clinical Centers Associate Director-Education network and selected, nationally-recognized clinical education experts outside VA. Results: An eight-member working group, interprofessional in composition, through multiple remote meetings has defined goals of the program, evaluated preliminary evidence addressing the clinical needs of older adults with pain, and posed ‘curious questions’ about the available large-scale data. The overarching goal is evidence-based needs assessment of gaps in education about pain in older adults, with purposeful attention to risks of healthcare inequities for older adult women, Veterans, persons of color, those at socioeconomic disadvantage, and caregivers. Conclusions: Interprofessional collaboration is effective in framing a broad needs assessment regarding pain and common comorbidities in older adults with the intent of meeting the educational needs of clinical trainees. More study is needed.

